# Autoimmunity and Extrahepatic Manifestations in Treatment-Naïve Children with Chronic Hepatitis C Virus Infection

**DOI:** 10.1155/2012/785627

**Published:** 2012-05-08

**Authors:** Giuseppe Indolfi, Elisa Bartolini, Biagio Olivito, Chiara Azzari, Massimo Resti

**Affiliations:** ^1^Paediatric and Liver Unit, Meyer Children University Hospital of Florence, Italy; ^2^Immunology Unit and Immunology Laboratory, Meyer Children University Hospital of Florence, Department of Sciences for Woman and Child's Health, University of Florence, Florence, Italy

## Abstract

Hepatitis C virus (HCV) infection has been associated with autoimmunity and extrahepatic manifestations in adults. Few data are available on these topics in children. Nonorgan specific auto-antibodies development is part of the natural course of chronic hepatitis C in children. Smooth muscle autoantibody is the most common autoantibody found, while liver-kidney microsomal type-1 antibody positivity is the most peculiar autoimmune feature of children with HCV infection. The clinical significance of non-organ specific autoantibodies in the course of paediatric chronic hepatitis C is still debated. Autoantibody positivity can be considered neutral for most patients, while it can be associated with negative connotations for others, especially those positive for liver-kidney microsomal type-1 autoantibody. Subclinical hypothyroidism but not autoimmune thyroiditis has been demonstrated in HCV infection in children, while only few cases of HCV-associated membranoproliferative glomerulonephritis have been described. Single reports are available in the literature reporting the anecdotal association between chronic hepatitis C and other extrahepatic manifestations such as myopathy and opsoclonus-myoclonus syndrome. Despite the low incidence of extrahepatic manifestations of chronic hepatitis C in children, overall, available data suggest a careful monitoring.

## 1. Introduction

Since its discovery in 1989 [1], hepatitis C virus (HCV) has been associated with autoimmunity and extrahepatic manifestations [2]. Data on these topics in children are scarce, but the incidence of extrahepatic manifestations is overall lower in children with chronic hepatitis C when compared to adults [2–6]. The purpose of the present article is to summarize the current knowledge on autoimmunity and extrahepatic manifestations in treatment-naïve children with chronic HCV infection.

## 2. Nonorgan Specific Autoantibodies

Nonorgan specific autoantibodies (NOSAs) development is considered part of the natural course of chronic HCV infection in children [3–6]. Different mechanisms have been implicated in the development of NOSAs during chronic hepatitis C [7]. The high prevalence of NOSAs in adults is considered the clear evidence of the altered immune system homeostasis in chronically infected patients. The characteristic lymphotropism of HCV could be one of the bases of the increased production of autoantibodies. It has been hypothesized that HCV interacting with B lymphocytes can lower the B-cell activation threshold favouring autoantibodies production, and that HCV, such as other hepatitis viruses, triggers autoimmune response via a molecular mimicry mechanism. Molecular mimicry originates when the target of the immune response to a microorganism shares similarities with a “self” antigen, and the original antimicrobial immune response becomes cross-reactive with the “self,” that is, autoimmune. By a complementary mechanism, HCV can induce cellular injury determining the release of “self” antigens that are normally protected from the immune system but when released are able to elicit an autoimmune response [8, 9].

The prevalence of NOSAs in children with chronic HCV infection has been investigated in few studies, and wide ranges of positive results have been found (Table 1) [3–6]. The heterogeneity of the prevalence estimations among different studies is due probably to technical differences in the laboratory methods used and to the fluctuating behaviour of autoantibodies. For these reasons studies based on determinations of NOSAs on serial samples [4], using lower dilution thresholds of positivity [4, 5] and more sensitive laboratory methods [4], had results higher than those based on single point determinations [3, 5], using higher thresholds of positivity [3, 6] and less sensitive methods.

Firstly, in 1996 Bortolotti et al. [3] analyzed the prevalence of NOSAs in forty Italian children with chronic HCV infection. About one third of the children studied had circulating NOSAs smooth muscle autoantibody (SMA) being the most common autoantibody found. Interestingly, in this cohort, patients with liver-kidney microsomal type-1 (LKM-1) autoantibody were more often infected by HCV genotypes 1 and 2, while no difference was found between autoantibodies positive and negative cases with respect to clinical features, *γ*-globulins levels, and liver histology. Few years later, in 1998 Gregorio et al. [4] in a cohort of fifty-one children with chronic infection using a lower dilution threshold of positivity (1 : 10) and a homemade substrate for indirect immunofluorescence found an higher prevalence of autoantibodies positivity (65%). SMA was again the most common autoantibody detected being present in 51% of the patients. Twenty-nine percent of the patients enrolled were positive for antibodies to gastric parietal cells [4]. Muratori et al. in 2003 [5] found NOSAs in 34% of forty-seven children, and they did not find any difference in clinical, virological, and biochemical parameters between NOSAs-positive and -negative children. Confirming previous findings in adults, NOSAs negative children in this cohort had significantly higher HCV viral load than those with NOSAs. As a possible explanation the authors speculated that the unbalanced immune reaction determining autoimmunity creates a cytokine environment which hampers viral replication [5]. In all the studies examined NOSAs titres were usually lower, and the antigenic specificity by indirect immunofluorescence was different than that usually found in children with autoimmune hepatitis (AIH) [3–5]. Antinuclear antibodies (ANA) presented with a speckled rather than homogeneous pattern and SMA reacted only with vessels, sparing glomerular and tubular structures which are additional targets in AIH type 1. Not all the children studied presented LKM-1 positivity reacting with the cytochrome P45IID6 (CYP2D6) that is the common target of LKM-1 autoantibody in type 2 AIH.

In the studies by Gregorio and Muratori [4, 5] children with chronic hepatitis C were enrolled together with children with chronic hepatitis B and with other chronic liver diseases as controls. Overall, NOSAs were more common in children with chronic hepatitis C than in children with other liver disorders of similar severity [4, 5], suggesting that the presence of autoantibodies is not just a consequence of the chronic liver damage. It is noteworthy that LKM-1 reactivity was not found in any of the controls suggesting that LKM-1 positivity, even if not the most common, was the most peculiar autoimmune feature of children with chronic hepatitis C. Furthermore, LKM-1 prevalence in HCV-infected children was consistently higher than that found in studies involving HCV-infected adults [2].

### 2.1. Clinical Significance of NOSAs

The clinical significance of NOSAs in the course of chronic hepatitis C is still debated [7]. The real challenge for clinicians and scientists is to understand whether and to what extent HCV-induced autoimmunity contributes to liver damage distinguishing two theoretically possible populations of children: (1) children with HCV infection and histologically silent HCV-induced autoimmunity and (2) children with HCV infection and HCV-driven autoimmune liver damage.

As first hypothesis, NOSAs reactivity in children with hepatitis C could be considered a simple consequence of hepatocellular damage without pathogenic significance. This hypothesis is supported by the observation that different children despite NOSAs presence and persistence have normal serum aminotransferase level, will not develop other features of autoimmunity such as increased *γ*-globulins levels, and usually have autoantibodies titres lower than that observed in AIH and with a different pattern as detected by indirect immunofluorescence [3–5]. On the other hand autoimmunity could have pathogenetic implications in liver damage of chronically infected children. Some data supporting this hypothesis are available. In adults some studies showed that the presence of autoantibodies was associated with elevated levels of *γ*-globulins, immunoglobulins, alkaline phosphatase, and *γ*-glutamil transpeptidase [10, 11]. Furthermore, LKM-1 positive HCV-infected children have been demonstrated to have a more advanced liver disease when compared with LKM-1 negative peers [12].

Positivity for LKM-1 autoantibody in chronically infected children appears to have important clinical implication in view of the significant biochemical deterioration observed in LKM-1 positive patients who developed marked increases in aminotransferase activity when treated with interferon-*α* [3–5, 12]. This is a hot topic given the recent approval of the combined treatment with pegylated interferon-*α* and ribavirin by FDA and EMA for children older than three years. It has been hypothesized that in LKM-1 positive children interferon-*α* may amplify the autoimmune response targeting CYP2D6 and thereby trigger acute LKM-1 mediated liver damage. It is important that treatment of LKM-1/HCV positive patients is decided after thorough investigations to exclude AIH. The issue of immunosuppressive therapy in these children is debated as it can improve clinical and biochemical parameters in selected patients, but it favours persistent HCV replication.

## 3. Thyroid

Few data are available regarding natural history of thyroid dysfunction and thyroid autoimmune disease in children with chronic HCV infection [4, 6, 13]. Gregorio et al. tested the presence of antithyroglobulin and antithyroperoxidase (TPOA) in chronic HCV positive children before and after treatment with interferon-*α* with no positive result [4]. Ghering et al. investigating thyroid function and prevalence of autoimmune phenomena in chronic HCV-infected children treated with interferon-*α*, found a strong correlation between treatment and emergence of thyroid antibodies [6]. Before treatment all children had normal thyroid function, one child had an isolate TSH elevation and a further one had borderline TPOA levels [6]. Indolfi et al. in a case-control study enrolling a cohort of untreated children with vertically acquired chronic HCV infection (*n* = 36), showed a high prevalence of subclinical hypothyroidism (11%) and of autoimmune thyroiditis (5.6%) [13]. Subclinical hypothyroidism was not related to length of infection, or to different HCV genotypes, but it was related to the presence of active liver disease. Subclinical hypothyroidism was not found in children with apparent virus clearance but only in those with chronic infection and persistent viraemia even though no correlation was found between development of subclinical hypothyroidism and levels of viraemia. No child with subclinical hypothyroidism in this study presented antithyroid autoantibodies suggesting that the mechanism of subclinical hypothyroidism in HCV-infected children is not antibody mediated [13], although autoimmunity cannot be excluded absolutely as in adults autoimmune thyroiditis without detectable circulating anti-thyroid antibody titres has been demonstrated and autoimmunity can be executed by autoreactive T cells also in the absence of detectable autoantibodies [14]. The possible role in the genesis of subclinical hypothyroidism during chronic HCV infection of the impaired hepatic metabolism of thyroid hormones [12] and of the thyroid infection by HCV [15] remains speculative.

## 4. Kidney

Membranoproliferative glomerulonephritis is the most common renal disease associated with HCV infection in adults [2]. To the best of our knowledge only three cases of HCV-associated membranoproliferative glomerulonephritis in children have been reported [16–18]. The specific pathogenesis of the glomerular injury caused by HCV infection is not known, but it is thought to result from the deposition of circulating immune complexes of HCV and anti-HCV on the glomerular capillary in the mesangium [2]. In one of the three children described, successful antiviral monotherapy with pegylated interferon-*α* resulted in HCV RNA clearance and disappearance of proteinuria [16].

## 5. Anecdotal Observations

Single reports are available in the literature reporting the anecdotal association between chronic hepatitis C and extrahepatic manifestations. Mohan et al. described a 15-year-old boy with diabetes, chronic HCV infection, and an inflammatory myopathy presumed to be associated with HCV infection [19]. The potential association between inflammatory myopathy and HCV infection has been infrequently reported in adult patients [20], and an autoimmune mechanism has been hypothesized. Ertekin and Tan reported a 9-year-old boy with progressive cerebellar ataxia, action myoclonus, palpebral flutter, vomiting, headache, and opsoclonus, diagnosed with opsoclonus-myoclonus syndrome [21]. This rare neurologic disorder characterized by multidirectional chaotic eye movements, myoclonus in the limbs, and ataxia may be associated with viral infections and for the first time with hepatitis C [21]. The exact immunopathogenesis of the disease is undefined [22], but as for other extrahepatic manifestations of HCV infection, the underlying mechanism was postulated to be immune system dysregulation.

## 6. Conclusions

The wide range of extrahepatic manifestations of HCV in adults suggests that HCV chronic infection should be considered a systemic disease. An important role in the development of extrahepatic manifestations of HCV is thought to be played by geographic, genetic, or environmental cofactors as well as by the disease activity [2]. Chronic hepatitis C is much different in children when compared to adults with regard to disease activity, response to treatment, and extrahepatic manifestations. Mixed cryoglobulinaemia, for example, is the most documented extrahepatic manifestation of HCV infection in adults and has never been described in children. Although discussed, the association between mixed cryoglobulinaemia and severe liver damage has been shown by different epidemiological studies [2, 23]. The natural history of chronic hepatitis in children is usually of a mild disease [24], and the low incidence of severe liver damage during childhood could be partly responsible for the absence of mixed cryoglobulinaemia in children. Despite the low incidence of extrahepatic manifestations in children, overall, available data suggest a careful monitoring.

## Figures and Tables

**Table 1:** Studies investigating the prevalence of nonorgan specific auto-antibodies in children with chronic hepatitis C.

	*n*	NOSAs	SMA	ANA	LKM-1	Dilution threshold of positivity	Sample/s used
Bortolotti et al. 1996 [3]	40	32.5%	17.5%	7.5%	10%	1 : 20	Single point determination
Gregorio et al. 1998 [4]	51	65%	51%	10%	8%	1 : 10	Sequential serum samples
Muratori et al. 2003 [5]	47	34%	17%	9%	15%	1 : 10	Single point determination
Gehring et al. 2006 [6]	39	8%	5%	—	2%	1 : 40	Two serum samples

Note: NOSAs, nonorgan specific autoantibodies; SMA, smooth muscle autoantibody; ANA, antinuclear antibody; LKM-1, liver kidney microsomal type-1 auto-antibody.
